# Immediate after-effects of robot-assisted gait with pelvic support or pelvic constraint on overground walking in healthy subjects

**DOI:** 10.1186/s12984-019-0506-z

**Published:** 2019-03-15

**Authors:** J. F. Alingh, V. Weerdesteyn, B. Nienhuis, E. H. F. van Asseldonk, A. C. H. Geurts, B. E. Groen

**Affiliations:** 1 Sint Maartenskliniek Research, PO BOX 9011, 6500 GM Nijmegen, The Netherlands; 2 Department of Rehabilitation, Donders Institute for Brain, Cognition and Behaviour, Radboud University Medical Center, Nijmegen, The Netherlands; 3 Department of Biomechanical Engineering, University of Twente, Enschede, The Netherlands

**Keywords:** Overground walking, Robotics, Pelvis, Kinematics, Spatiotemporal parameters

## Abstract

**Background:**

Recovery of walking is a primary rehabilitation goal of most stroke survivors. Control of pelvic movements is one of the essential determinants of gait, yet surprisingly, conventional robot-assisted gait trainers constrain pelvic movements. Novel robot-assisted gait trainers, such as LOPES II, are able to support pelvic movements during gait. The aim of this cross-over study was to investigate the immediate after-effects of pelvic support (PS) or pelvic constraint (PC) gait training with LOPES II on overground walking in healthy subjects.

**Methods:**

Thirteen able-bodied subjects (22.8 ± 2.1 years) participated in two 20-min gait training sessions with LOPES II; one with PS and one with PC. During the PS-training, the LOPES II actively guided the lateral displacement of the pelvis, while pelvic rotations were free. During the PC-condition, both lateral displacement and pelvic rotations were constrained and reduced to a minimum. The training sessions were separated by a 30-min resting period. Lateral displacement of the pelvis, hip and knee kinematics, and spatiotemporal parameters during overground walking were determined at baseline and immediately following the training using 3D gait analysis.

**Results:**

During the PS-condition in LOPES II the lateral pelvic displacement was significantly greater (105.6 ± 0 .5 mm) than during the PC-condition (10.8 ± 0 .7 mm; *p* < 0.001). Analysis of the first five steps of overground walking immediately following PC-condition showed significantly smaller lateral displacements of the pelvis (32.3 ± 12.0 mm) compared to PS-condition (40.1 ± 9 .8 mm; *p* < 0.01). During the first five steps, step width was significantly smaller after PC-condition (0.17 ± 0. 04 m) compared to PS-condition (0.20 ± 0.04 m; *p* = 0.01) and baseline (0.19 ± 0. 03 m; *p* = 0.01). Lateral displacement of the pelvis and step width post training returned to baseline levels within 10 steps. PC- nor PS-condition affected kinematics, gait velocity, cadence, stride length or stance time.

**Conclusions:**

In healthy subjects, robot-assisted gait training with pelvic constraint had immediate negative after-effects on the overground walking pattern, as compared to robot-assisted gait training with pelvic support. Gait training including support of the lateral displacement of the pelvis better resembles the natural gait pattern. It remains to be identified whether pelvic support during robot-assisted gait training is superior to pelvic constraint to promote gait recovery in individuals with neurological disorders.

## Background

Recovery of walking is an important rehabilitation goal for many stroke survivors [1]. Although many individuals after stroke regain some degree of walking capacity, the hemiparetic gait pattern is commonly characterized by spatiotemporal asymmetry, reduced walking speed, and impaired balance control [2, 3]. Furthermore, hemiparetic gait is associated with atypical pelvic movements [4]. Since control of pelvic movements is one of the essential determinants of gait [5], restoring the normal pelvic movement pattern seems a crucial target for gait training after stroke. Nowadays, robotic gait trainers are increasingly used for the rehabilitation of stroke survivors [6], as well as individuals with spinal cord injury [7] or cerebral palsy [8]. First-generation robotic gait trainers used for the rehabilitation of these individuals, however, generally impose restrictions to both lateral translations and rotations of the pelvis.

Restriction of pelvic movements imposed by a robotic gait trainer substantially influences the gait pattern. Indeed, in healthy adults, walking with restrictions of both lateral pelvic translations and rotations may result in narrower step width and excessive trunk rotations [9], shorter [10] or longer step length [9] and reduced range of motion of the lower limb joints [10]. In addition, restrictions of pelvic movements may yield increased activation of the adductor longus muscle [11], whereas no effect on gluteus medius activity was observed during stance [10, 11]. Furthermore, reduced pelvic range of motion during treadmill walking was shown to be retained during unconstrained treadmill walking [12]. These observations raise the question whether these adverse effects of restricted pelvic movements during robotic gait training might be transferred to unconstrained overground walking after robotic gait training. If such effects would occur, robotic gait training involving restricted pelvic movements might even be detrimental for relearning an optimal gait pattern after stroke. Conversely, adding degrees of freedom to the pelvis might enable patients to adopt a more normal gait pattern while walking in a robotic gait trainer [11]. Newly developed robotic gait trainers like the lower extremity powered exoskeleton LOPES [13, 14] and the latest version of the Lokomat (Lokomat Free-D module) allow more degrees of freedom at the pelvic level. In addition, some of these new generation robotic gait trainers can provide support of pelvic movements tailored to the individual patient’s need.

Although support of pelvic movements has the potential to improve the gait pattern during robotic gait training and its effect was shown to be retained during unconstrained treadmill walking [12], the immediate after-effects of either pelvic support or pelvic constraint on the unconstrained, *overground* gait pattern have not yet been studied. As overground walking more closely resembles walking in daily life than treadmill walking does, investigating the transfer of these pelvic conditions to overground walking is important. Therefore, the aim of the current study was to investigate the immediate after-effects of robot-assisted gait with either pelvic constraint or pelvic support on the first meters of overground walking in healthy subjects, thereby resembling the training conditions in the first-generation and new generation robotic gait trainers, respectively. Furthermore, the overground gait pattern after robot-assisted gait with pelvic constraint or pelvic support was compared with the normal overground gait pattern. It was hypothesized that restricting pelvic movements during robot-assisted gait would lead to reduced lateral pelvic translations during overground walking compared to robot-assisted gait with pelvic support and normal overground walking. Robot-assisted gait with pelvic support was expected to more closely resemble the lateral pelvic displacements of normal gait.

## Methods

### Participants

From April 2014 to July 2014 a total number of 14 healthy young adults were recruited in Nijmegen, the Netherlands, to participate in this study. Participants did not suffer from any injury or impairment interfering with balance and gait. All participants gave written informed consent. The study was designed following the Declaration of Helsinki. The study protocol (NL 42426.044.12) was approved by the Medical Ethical Committee Twente (Enschede, the Netherlands).

### Design

**Fig. 1:**
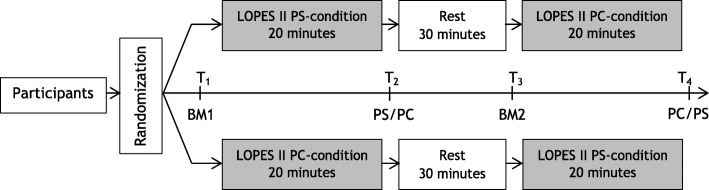
Study design. Before and after the 20-min walk in LOPES II a 3D-gait analysis was performed (baseline measurement 1 (BM1), baseline measurement 2 (BM2), pelvic support (PS), pelvic constraint (PC))

Participants were enrolled in a cross-over study, including two 20-min walking conditions in the robotic gait trainer LOPES II with either pelvic support (PS) or pelvic constraint (PC). Each LOPES II condition was preceded and followed by an overground 3D-gait analysis (Fig. 1).

### Materials

#### Robotic gait trainer

**Fig. 2:**
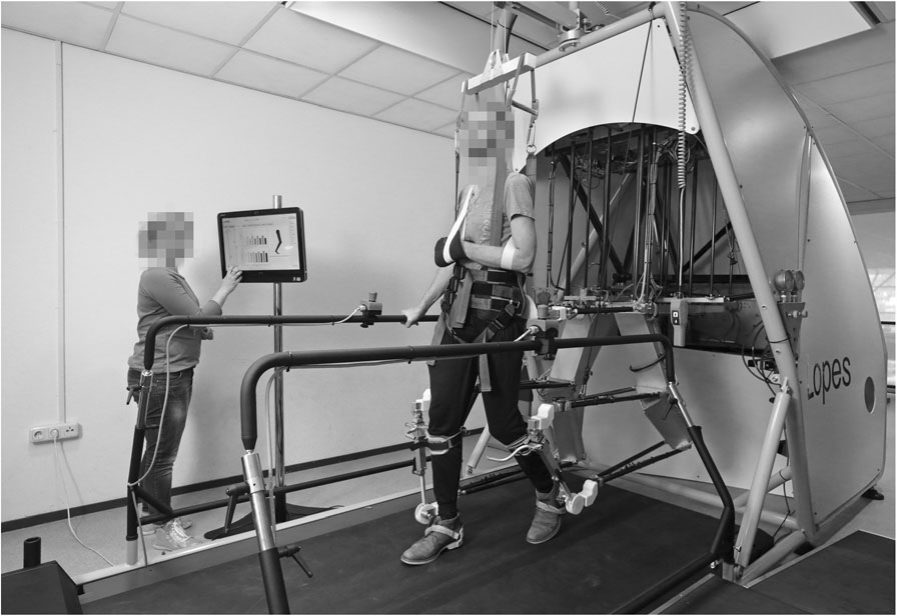
Robotic gait trainer LOPES II

The LOPES II is a robotic gait trainer, combined with a treadmill and body weight support system (Fig. 2). LOPES II has eight powered degrees of freedom, actuating knee flexion/extension, hip flexion/extension, hip adduction/abduction, and pelvic translations in the lateral and anterior/posterior directions. In addition, the robot allows free motion of pelvic rotations, hip and foot endorotation/exorotation, and ankle plantarflexion/dorsiflexion and inversion/eversion. Together, its settings allow pelvic movements to be either supported or mechanically constrained. The level of support is adjusted for each individual, as LOPES II controls the interaction forces during gait training. The applied forces are calculated from the deviation of the actual movement from the predefined gait trajectory, and the set level of guidance force. The level of guidance force can be set in two parts: the general guidance force level is set for all subtasks of walking, and on top of that the specific guidance force level can be adjusted for each specific gait subtask, such as ‘weight shift guidance’. Together, LOPES II applies joint torques to re-direct the deviations in pelvis and limb movements from the participant’s gait trajectory towards the predefined, desired gait trajectory. The applied predefined gait trajectories are derived from the gait pattern of healthy walkers and adjusted to the individual’s walking velocity and participant’s height. For a detailed description of the LOPES II and its control we refer to a publication by Meuleman et al. [14].

**Fig. 3:**
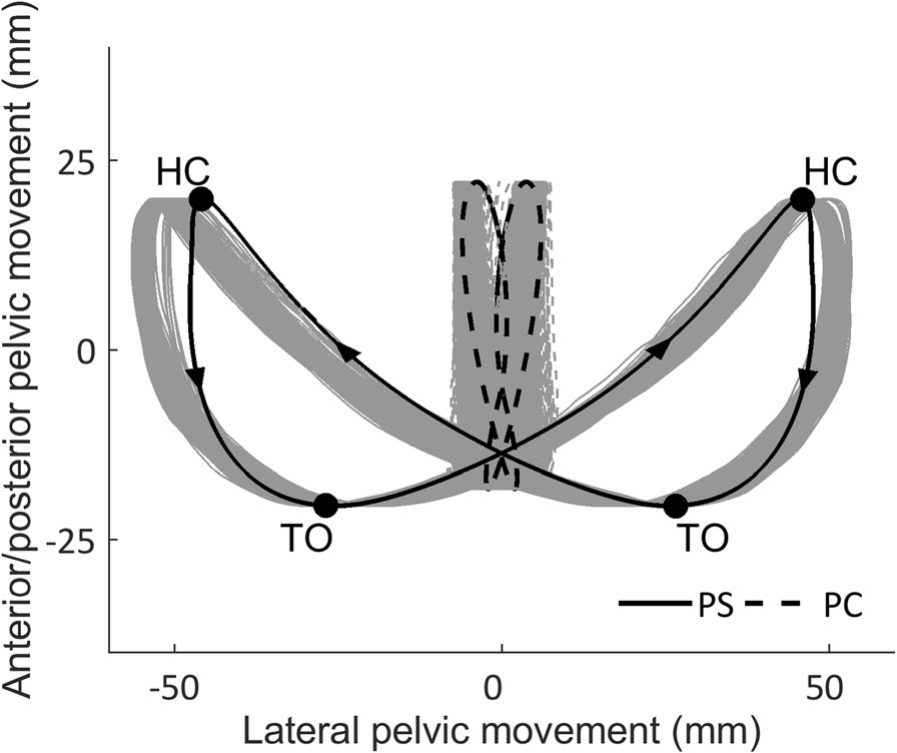
Typical example of imposed (black line) and actual (grey line) pelvic movements in the transverse plane (X-Y) during gait in LOPES II for the pelvic support (PS) and pelvic constraint (PC) conditions. Pelvic position was determined by the midpoint between the hip joints. The pelvic movements were corrected for the displacement in the line of progression. Arrows indicate the direction of the pelvic displacements. The markers represent the moment of heel contact (HC) and toe off (TO)

The general guidance force and lateral translations and rotations of the pelvis varied between the two LOPES II conditions. During the PS-condition the lateral pelvic translations were guided towards the imposed pre-defined trajectory (low level of general guidance: 15 Nm/rad; high level of weight shift guidance: 20 N/mm; large weight shift amplitude: 53 mm), while pelvic rotations were left free. During the PC-condition both the lateral pelvic translations and pelvic rotations were constrained, reducing the participants’ pelvic movements to a minimum (high level of general guidance: 1500 Nm/rad; high level of weight shift guidance: 20 N/mm; small weight shift amplitude: 5.3 mm). Figure 3 shows the imposed and actual pelvic movements while walking in LOPES II during both the PS- and PC-conditions. During the PS- and PC-condition, the LOPES II recorded pelvic position, segment angles (for calculating kinematics), spatiotemporal gait parameters and interaction forces between the robot and the participant (f_s_ = 100 Hz). Participants walked at a standardized gait speed of 0.55 m/s, a speed that is also used during robotic gait in our ongoing intervention study in people in the subacute phase after stroke. Body weight support was set at 10% of the total body mass to carry the load of the system.

#### 3D-gait analysis

To assess the immediate after-effect of the LOPES II condition on the overground gait pattern, a 3D-gait analysis was performed before and immediately after each LOPES II condition. Twenty reflective markers (14 mm) were attached to the participants’ skin, according to the Plug-In-Gait Lower body model (Plug-in-Gait, Vicon Motion Systems Ltd., Oxford, UK). Marker positions were indicated on the skin to ensure fast and equal marker placement across the gait analyses. The position of the reflective markers was registered by ten infrared cameras (f_s_ = 100 Hz; Vicon mX 1.7.1, Oxford Metrics, UK). Participants walked at a self-selected comfortable speed across a 6-m walkway. A single walk across the walkway was defined as a trial. A total of five trials were collected for each participant at every gait assessment, recording at least five steps walked within the capture volume per trial.

#### Procedure

A 3D-gait analysis (Baseline Measurement 1) was performed at the start of the experiment to determine the overground gait pattern (T1). Participants were then installed in LOPES II and completed a 20-min walking condition with either supported or constrained pelvic movements. Participants walked without handrail support and were instructed to follow the pre-defined trajectory of the LOPES II. After completing a LOPES II condition, participants were transferred to the gait laboratory in a wheelchair to ensure no more than 3 steps of walking before the start of the second gait analysis. The reflective markers were reattached to the skin and a new analysis was performed within 5 min after the end of the LOPES II condition (T2). After completing this second 3D-gait analysis a 30-min break was allowed. Thereafter, the procedure was repeated for the second LOPES II condition, yielding Baseline Measurement 2 (T3) followed by the fourth 3D-gait analysis (T4).

#### Data analysis

Data collected by LOPES II was used to determine the kinematics and spatiotemporal gait parameters during the last 40 strides of walking in the PC- and PS-conditions. To determine participant’s final level of adaptation to the applied condition, the root mean square (RMS) of the interaction forces in the mediolateral direction at the pelvis were calculated for the last 40 strides of walking in each condition in LOPES II. The Vicon Plug-In-Gait model and software were used to calculate the kinematics and spatiotemporal gait parameters for each trial of the 3D-gait analysis. Data was further analyzed using custom-written software (MATLAB, Mathworks Inc., Natick, MA, USA). The lateral pelvic displacement (LPD) during robot-assisted gait and unsupported overground walking was defined as the absolute peak-to-peak displacement (mm) of the pelvis perpendicular to the walking direction during each stride. In addition, we determined range of motion of the knee and hip joint in the sagittal plane and spatiotemporal parameters including single-support time (%), stride length (m), step width (m), gait velocity (m/s), and cadence (steps/min). Average values were calculated for all variables per trial.

#### Statistics

The statistical analysis was performed using Stata software (TX StataCorp LP 2013, version 13). The normal distribution of the LPD, interaction forces, spatiotemporal parameters, and kinematics was tested using a Shapiro-Wilk test. Thereafter, two-sided paired-samples *t* tests were performed to examine differences in LPD, interaction forces, spatiotemporal parameters, and kinematics *during* gait in LOPES II between the last 40 strides of walking in the PS- and PC-conditions. Next, one-way repeated measures analyses of variance with ‘Condition’ (PS-condition, PC-condition, and Baseline Measurement) as within-subjects factor were performed to determine differences in LPD, spatiotemporal parameters, and kinematics for the first trial of overground walking immediately *after* the PS- and PC-condition. Since no significant differences were found between the LPD, spatiotemporal parameters, or kinematics of Baseline Measurements 1 and 2, the mean value per trial was used as a reference (Baseline Measurement). Post-hoc paired-samples *t* tests with Bonferroni correction were applied to correct for multiple comparison (*p* < 0.017). To evaluate the persistence of any after-effect, the effects of ‘Condition’ (PS-condition, PC-condition, Baseline Measurement) and ‘Time’ (Trial 2–5) on LPD, spatiotemporal parameters, and kinematics were determined using a two-way repeated measures analysis of variance. The correlation between the interaction forces measured during the last 40 strides of walking in each condition in LOPES II, and the change in overground LPD from baseline to the post-measurement was calculated using a Pearson’s correlation coefficient. In addition, the correlation between the change in LPD from baseline to walking in the robot during the PS- and PC-condition, and the presence of after-effects was calculated using a Pearson’s correlation coefficient. The significance level was set at *p* < 0.05 for all tests, unless mentioned otherwise.

## Results

Of the 14 healthy adults included, 13 participants completed all assessments (men/women 2/11, age 22.8 ± 2.1 years; length 1.78 ± 0.06 m, weight 71.42 ± 8.63 kg; mean ± SD). One participant was excluded from the analysis, because the PC-condition was not completed due to technical problems.

### Lateral pelvic displacement

During robot-assisted gait with pelvic support, the LPD reached an average displacement of 105.6 ± 0.5 mm, which was significantly greater than the LPD during the PC-condition (10.8 ± 0.7 mm; t_(12)_ = 115.28, *p* < 0.001; see Table 1). The RMS of the interaction forces during walking in LOPES II was significantly smaller during the PS-condition (RMS: 35.2 ± 11.0 N) compared to the PC-condition (RMS: 73.8 ± 14.7 N; t_(12)_ = 6.821, *p* < 0.001).

**Table 1:** Mean (±SD) values for lateral pelvic displacement (LPD), spatiotemporal gait parameters, and range of motion of the hip and knee joint in the sagittal plane during walking in the robotic gait trainer LOPES II averaged across participants and across strides within the pelvic support (PS) and pelvic constraint (PC) conditions

	PS	PC
LPD (mm)	105.6 (0.5)	10.8 (0.7)*
Gait velocity (m/s)	0.55 (0.01)	0.55 (0.01)
Cadence (steps/min)	62.8 (3.8)	64.8 (3.2)
Stride length (m)	1.11 (0.09)	1.04 (0.05)*
Single-support time (% gait cycle)	58.91 (1.44)	56.77 (0.68)*
Step width (m)	0.16 (0.01)	0.15 (0.01)*
Range of motion		
Hip (degrees)	34.9 (0.9)	34.4 (0.1)
Knee (degrees)	55.0 (0.7)	51.3 (0.2)*

*significantly different from PS, *p* < 0.001

**Fig. 4:**
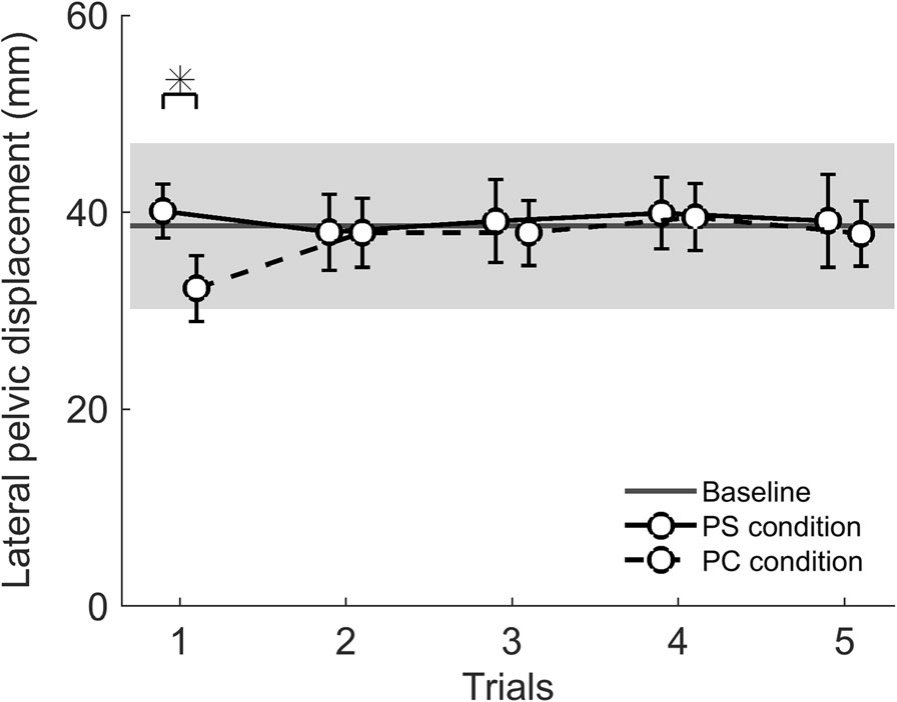
Average lateral pelvic displacement (LPD) recorded during overground walking (trials 1–5) for both baseline measurements, the pelvic support (PS) and pelvic constraint (PC) conditions (*N* = 13). The 95% confidence interval of the baseline LPD across trials is represented by the grey area. Markers represent the LPD values recorded after the PS- (black solid line) and PC-conditions (black dashed line). Error bars indicate the standard error of the mean

**Fig. 5:**
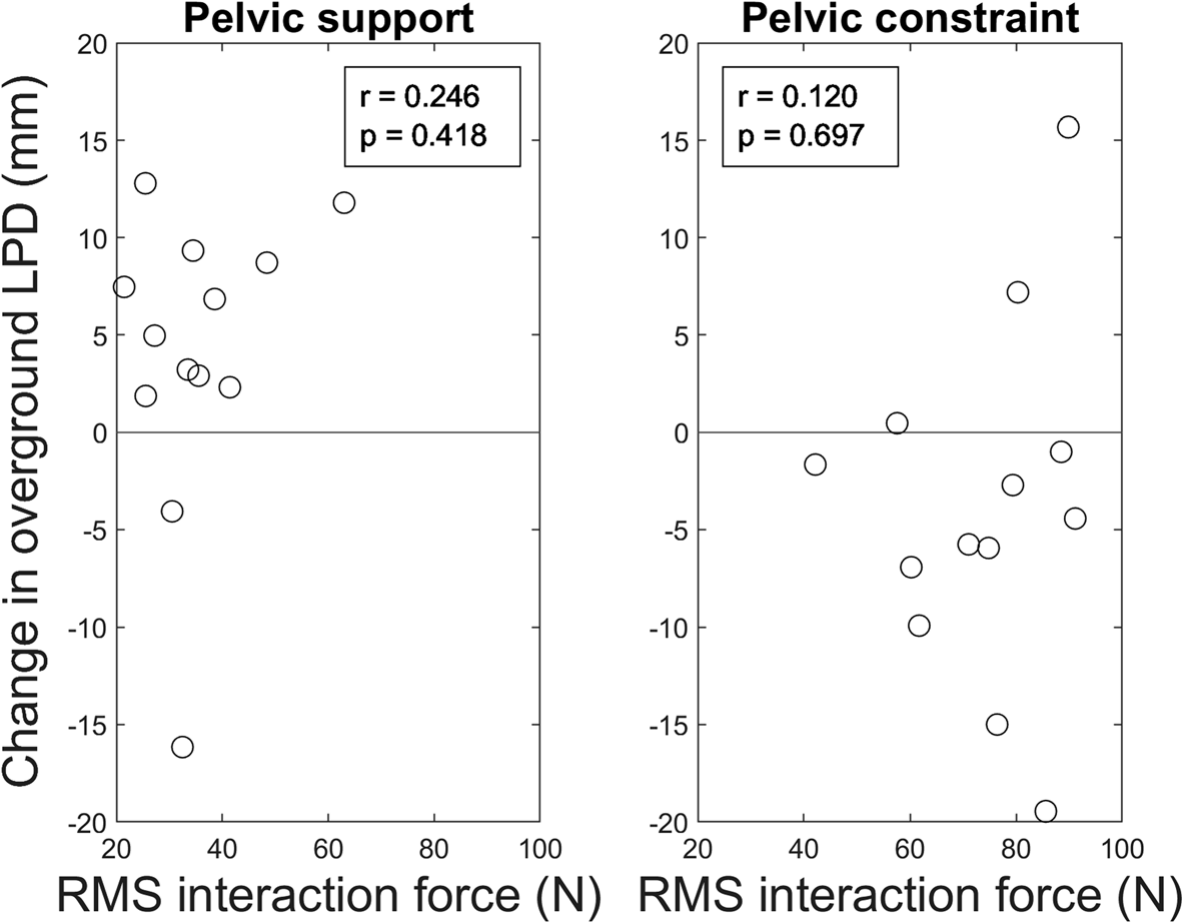
Root mean square (RMS) of the interaction force experienced by each participant during walking in LOPES II in the pelvic support (left panel) and pelvic constraint condition (right panel) plotted against the change from baseline in lateral pelvic displacement (LPD) during overground walking in the respective condition. Accompanying correlation coefficients and *p*-values are provided. Larger mean RMS interaction forces indicate less adaptation to the movement pattern imposed by the robot. A positive value for the change in LPD indicates that LPD had increased relative to baseline

LPD values recorded during the overground gait analyses are shown in Table 2. In trial 1, the LPD was significantly different between conditions (F_(2, 12)_ = 5.350, *p* = 0.039). Post-hoc analyses showed that LPD in trial 1 was significantly smaller after the PC-condition compared to the PS-condition (t_(12)_ = 3.059, *p* = 0.009; see Fig. 4). Compared to baseline, trial 1 after the PC-condition resulted in slightly smaller LPD values and after the PS-condition in slightly larger LPD values, but these differences did not reach significance (t_(12)_ < 1.901 *p* > 0.082). In addition, no main or interaction effects of Condition or Time were found for trials 2–5 (F_(6,132)_ < 0.420, *p* > 0.525). There was no correlation between the individual interaction forces during walking in LOPES II and the presence of after-effects in LPD in either condition (PS: *r* = 0.246, *p* = 0.418; PC: *r* = 0.120, *p* = 0.697; see Fig. 5). In addition, there was no correlation between the difference in LPD during walking in the robot and overground walking at baseline, and the presence of after-effects following the PS- (*r* = 0.41, *p* = 0.159) or PC-condition (*r* = 0.28, *p* = 0.348).

**Table 2:** Mean (±SD) values for lateral pelvic displacement (LPD), spatiotemporal gait parameters, and kinematics during overground walking averaged across participants and across steps within the trial for the baseline (BM) and pelvic support (PS) and pelvic constraint (PC) conditions

	Trial 1			Trial 2		Trial 3		Trial 4		Trial 5	
	BM	PS	PC	PS	PC	PS	PC	PS	PC	PS	PC
LPD (mm)	36.1 (10.2)	40.1 (9.8)	32.3 *^a^ (12.0)	37.9 (13.9)	37.9 (12.6)	39.1 (15.1)	37.9 (11.8)	39.9 (13.1)	39.5 (12.2)	39.1 (16.9)	37.8 (11.8)
Gait velocity (m/s)	1.36 (0.14)	1.30 (0.17)	1.32 (0.16)	1.34 (0.19)	1.14 (0.45)	1.33 (0.18)	1.35 (0.16)	1.33 (0.19)	1.33 (0.16)	1.32 (0.16)	1.36 (0.16)
Cadence (steps/min)	107.5 (5.0)	104.8 (7.9)	106.5 (5.7)	105.2 (7.1)	105.4 (4.7)	106.4 (6.9)	107.4 (5.1)	104.4 (6.3)	106.2 (5.2)	106.2 (6.8)	107.7 (4.4)
Stride length (m)	1.50 (0.14)	1.49 (0.13)	1.48 (0.14)	1.50 (0.14)	1.46 (0.15)	1.50 (0.14)	1.49 (0.13)	1.51 (0.15)	1.49 (0.15)	1.49 (0.13)	1.49 (0.14)
Single-support time (% gait cycle)	62.74 (2.15)	63.26 (1.30)	62.67 (1.90)	63.22 (1.68)	63.21 (1.31)	62.71 (2.16)	62.23 (2.00)	63.19 (1.52)	62.48 (1.62)	62.44 (1.77)	61.85 (1.79)
Step width (m)	0.19 (0.03)	0.20 (0.04)	0.17 *^b^ (0.04)	0.20 (0.03)	0.17 (0.03)	0.19 (0.03)	0.18 (0.03)	0.19 (0.02)	0.19 (0.04)	0.19 (0.02)	0.19 (0.04)
Range of motion											
Hip (degrees)	50.0 (3.6)	50.4 (4.9)	49.4 (3.6)	49.6 (4.4)	49.4 (6.2)	52.0 (6.1)	51.4 (5.3)	49.8 (4.8)	49.2 (4.1)	51.9 (6.0)	53.3 (3.8)
Knee (degrees)	63.8 (2.4)	62.6 (3.4)	62.9 (2.4)	62.3 (4.0)	63.0 (2.9)	62.8 (4.7)	63.0 (4.6)	62.6 (3.2)	62.6 (2.3)	63.2 (3.6)	63.6 (4.8)

*^a^ significantly different from PS in trial 1, *p* = 0.009

^*b^significantly different from PS and BM in trial 1, *p* = 0.013 and *p* = 0.013 respectively

### Spatiotemporal parameters and kinematics

During robot-assisted gait with pelvic constraint, the step length, single support time and step width were significantly smaller than during the PS-condition (t_(12)_ = 2.229, *p* < 0.05; t_(12)_ = 5.449, *p* < 0.001; and t_(12)_ = 6.499, *p* < 0.001 respectively). Knee range of motion was slightly smaller during walking in LOPES II in the PC-condition than in the PS-condition (t_(12)_= 4.550, *p* < 0.001; see Table 1).

Average values for the overground spatiotemporal gait parameters recorded during the baseline measurements and after the PS- and PC-conditions are reported in Table 2. In trial 1, the step width was significantly different between conditions (F_(2, 12)_ = 6.340, *p* = 0.027). In line with the smaller LPD following the PC-condition, the post-hoc analysis showed that step width was significantly smaller after the PC-condition compared to the PS-condition (t_(12)_ = 2.925, *p* = 0.013) and baseline (t_(12)_ = 2.897, *p* = 0.013). No main or interaction effects of Condition or Time were found for step width for trial 2–5, or for gait velocity, cadence, stride length, or single-support time for any trials. No main or interaction effects of Condition or Time were observed for hip or knee range of motion during trial 1 and trial 2–5 of overground walking.

## Discussion

The aim of the current study was to investigate the immediate after-effect of robot-assisted gait with pelvic support or pelvic constraint on overground walking in healthy adults. As hypothesized, we found that applying a pelvic constraint (PC) reduced the lateral pelvic displacement during the first steps of overground walking in healthy adults when compared to overground walking immediately after the pelvic support (PS) condition. The after-effect in LPD following the PC- compared to PS-condition lasted no longer than one trial (i.e. five steps), which was followed by comparable LPD values across conditions in trials 2–5. In agreement with the reduced LPD in the first trial, the step width also decreased following the PC-condition. Other spatiotemporal gait parameters or kinematics during overground walking were not altered after robot-assisted gait with either pelvic support or pelvic constraint.

Even though participants were able to change their pelvic movements to the applied trajectory in both conditions, the smaller interaction forces measured during the PS-condition reflected a higher level of adaptation to the robot compared to the PC-condition. In the PS-condition, the interaction forces were only marginally higher than those observed for a ‘minimal impedance’ walking condition resembling free walking (RMS interaction force: 33.5 N; unpublished observations). This suggests that participants actively moved their pelvis along with the pattern imposed by LOPES II, instead of being passively guided by (i.e. pushed) or working against the robot. In the PC-condition, the larger interaction forces raise the question as to whether the participants (partly) reduced their active pelvic movements to the imposed constraint; or whether they were trying hard to move their pelvis and actively worked against the robotic constraint. The answer to this question can only be speculated upon from the direction of the observed after-effects. Previous adaptation studies in which participants had to adapt to a perturbing force while performing a movement, involved generating opposing forces and joint torques to perform the movement correctly. In these experiments, an overshoot of the movement was typically observed after removal of the perturbation [15–17]. Hence, the LPD ‘undershoot’ following the PC-condition in the present study suggests that our participants did not actively work against the robotic constraint, but indeed (partly) adapted to the robotic constraint.

The finding that walking in the robotic gait trainer with constrained lateral translation and rotation of the pelvis tends to affect the *subsequent* overground gait pattern in healthy adults adds to previous studies reporting altered gait *during* walking with a pelvic constraint [9, 10] and following walking with a constraint of the lower extremity on a treadmill [15–17] or pelvis [12, 18]. In agreement with our findings, these studies showed decreased range of motion at the knee and hip joints [10] and smaller step widths [9, 12] during walking with constrained lateral translations and rotations of the pelvis. In the present study, we also demonstrate a significant after-effect on overground LPD and step width following walking in a robotic gait trainer with a pelvic constraint. The reduced LPD during overground walking following the PC-condition is in line with previous studies conducted on a treadmill. These studies showed that constrained or supported pelvic translations resulted in reduced or enlarged pelvic movements and step width during unconstrained treadmill walking, respectively [12, 19]. The smaller step width observed in our study during overground walking might be due to the applied pelvic constraint in combination with the decreased step width *during* walking in the robotic gait trainer.

Although present, the observed after-effect on LPD and step width of walking with pelvic constraint in the present study was relatively small and quickly disappeared over time. The relative duration and size of the observed after-effect was modest compared to previous results from locomotor adaptation studies in healthy subjects. A review by Reisman et al. [20] reported after-effects ranging from 20 strides to 30 min following 5–180 min of walking in an experimental condition. This discrepancy may be due to the varying duration and type of applied conditions. For example, short after-effects (13–20 steps) were observed after 188 steps of walking in a robotic gait trainer with resistance applied to the hip and knee [21], whereas 10 min of split-belt training induced more persistent after-effects, lasting up to 6 min [22]. Based on the duration of the currently applied pelvic condition, a longer after-effect might have been expected. However, as after-effects are supposed to be greatest when the training and experimental conditions are similar [23–25], the transition from the robotic gait trainer to overground walking at a higher velocity might have reduced the observed after-effect. The difference in velocity between walking overground and in the LOPES II may be considered a limitation of the study.

Another limitation of the current study is the difference between the PC- and PS-condition in the general guidance force applied to the lower limbs. Yet, we purposely chose to apply the maximum general guidance force during the PC-condition, as these settings closely resemble the procedures used by first-generation robotic gait trainers [26]. On the other hand, newer robotic gait trainers can support the pelvic movements and also allow adjusting the guidance levels to the individual patient’s needs [14]. As we aimed to resemble the training conditions in older versus newer robotic gait trainers, we selected lower levels of general guidance force during the PS-condition. Because of this deliberate difference between the PS- and PC-conditions, we cannot rule out the possibility that the greater general guidance force in the PC-condition may have influenced (i.e. increased or decreased) the observed after-effects in LPD and step width during overground walking.

The reduced LPD and accompanying decrease in step width during overground walking in healthy controls following the pelvic constraint condition may have implications for robot-assisted gait training in people with neurological disorders (e.g. stroke, spinal cord injury). First-generation robotic gait trainers used for rehabilitation have limited control over the pelvic movements and constrain the pelvis during walking [11]. The present results indicate that these pelvic restrictions have the potential to undesirably carry over to overground walking. This effect was very short-lived in our healthy young participants after only 20-min of walking with pelvic constraint, yet repeated exposure and adaptation to a perturbation may result in longer lasting or even permanent changes in motor behavior [27]. Results from classical perturbation studies show that individuals with neurological disorders adapt differently to the applied perturbation compared to healthy controls. These individuals seem to be less capable to adapt [28], need more time to adapt [29], and their adaptation may vary greatly between individuals [30]. As pelvic constraint interferes with frontal plane balance control, we expect that constrained pelvic movements have a negative impact on the overground walking pattern of individuals with impaired gait due to a neurological disorder. In particular, individuals suffering from severe balance problems in the frontal plane and individuals with an ataxic gait pattern may experience such a negative impact of pelvic constraint on overground walking. It remains for future research to identify if individuals with impaired gait due to neurological disorders adapt to applied pelvic constraints during robotic gait training, and whether this may have a negative impact on the overground walking pattern. And if so, whether pelvic support during robot-assisted gait training may be superior to a pelvic constraint for promoting gait recovery in individuals with neurological disorders.

## Conclusions

This cross-over study shows that robot-assisted gait training with pelvic constraint has an immediate negative after-effect on the overground walking pattern in healthy subjects as compared to robot-assisted gait training with pelvic support. The after-effects were relatively small and short-lived, yet the effect of applying pelvic constraint or support during robot-assisted gait training in people with neurological gait impairments remains to be determined.

## Abbreviations

BM: Baseline measurement
HC: Heel contact
LPD: Lateral pelvic displacement
PC: Pelvic constraint
PS: Pelvic support
RMS: Root mean square
TO: Toe off
